# Draft genome sequence of *Streptomyces tunisialbus* DSM 105760^T^

**DOI:** 10.1007/s00203-020-01913-z

**Published:** 2020-05-30

**Authors:** Ameni Ayed, Daniel Wibberg, Imène Zendah el Euch, Marcel Frese, Ferid Limam, Norbert Sewald

**Affiliations:** 1 Laboratory of Bioactive Substances, Biotechnology Center of Borj-Cedria (CBBC), BP-901, 2050 Hammam-Lif, Tunisia; 2 Center for Biotechnology (CeBiTec), Bielefeld University, 33501 Bielefeld, Germany; 3 Organic and Bioorganic Chemistry, Faculty of Chemistry, Bielefeld University, 33615 Bielefeld, Germany

**Keywords:** *Streptomyces tunisialbus*, Draft genome, AntiSMASH 5.1.2, Secondary metabolites

## Abstract

*Streptomyces* strains are well known as promising source of bioactive secondary metabolites, important in ecology, biotechnology and medicine. In this study, we present the draft genome of the new type strain *Streptomyces tunisialbus* DSM 105760^T^ (= JCM 32165^T^), a rhizospheric bacterium with antimicrobial activity. The genome is 6,880,753 bp in size (average GC content, 71.85%) and encodes 5802 protein-coding genes. Preliminary analysis with antiSMASH 5.1.2. reveals 34 predicted gene clusters for the synthesis of potential secondary metabolites, which was compared with those of *Streptomyces varsoviensis* NRRL ISP-5346.

## Introduction

Since the discovery of streptomycin in 1944 from *Streptomyces griseus,* the first antibiotic isolated from bacteria (Schatz et al. 1944), Actinobacteria have obtained large attention from the scientific community, mainly due to their ability in producing a wide spectrum of bioactive compounds with antimicrobial and antitumor activities (Demain 2014; Barka et al. 2016). Nowadays, this class provided more than 65% of antibiotics and antifungals used in medicine; including over 10,000 bioactive compounds that were produced by the members of the genus *Streptomyces* (Bérdy 2005, 2012; De Lima et al. 2012). This genus, proposed for the first time by Waksman and Henrici (1943), belong to the Gram-positive bacteria and is ubiquitous in nature (Kämpfer 2012). The classification of *Streptomyces* species is generally carried out using a polyphasic taxonomic approach, including chemotaxonomic, phenotypic, and genotypic characteristics (Anderson and Wellington 2001). Currently (Status April 2020), about 841 species of the genus *Streptomyces* with valid names have been described and published so far (https://www.bacterio.net/streptomyces.html). Thus, several studies have been conducted to find new strains isolated from extreme habitats (Tiwari and Gupta 2012; Goodfellow 2013; Zhang et al. 2016). To cope with the major health problem of antibiotic-resistant microorganisms, many laboratories have focused on the discovery of new bioactive molecules produced from new species belonging to this genus. In fact, whole-genome sequencing (wgs) have opened new potential for better exploitation of useful secondary metabolites produced by this genus (Harrison and Studholme 2014; Lee et al. 2018; Ward and Allenb 2018). This fundamental technology was supported by the use of new bioinformatics tools like antiSMASH, which adds several new features, including prediction of gene cluster (Blin et al. 2017).

In this study, the genome of a new strain *Streptomyces tunisialbus* DSM 105760^T^, which produces a broad spectrum of secondary metabolites with antibacterial and antifungal effects (Ayed et al. 2018), was sequenced. The genome sequence of *S. tunisialbus* was established to broaden the genomic basis for functional and comparative analyses focusing on secondary metabolite biosynthesis clusters of the strain.

## Materials and methods

The genomic DNA was extracted from a culture grown 3 days in ISP2 medium (Shirling and Gottlieb 1966), using cetyltrimethylammonium bromide (CTAB)-based method (Hopwood 2000). The quality of the DNA was assessed by gel-electrophoresis, and its quantity was determined by a fluorescence-based method using the Quant-iT PicoGreen dsDNA kit (Invitrogen) and the Tecan Infinite 200 Microplate Reader (Tecan Deutschland GmbH). For sequencing of the *S. tunisialbus* DSM 105760^T^ genome, 4 μg of purified DNA was used to generate a paired library (TruSeq DNA PCR-Free Library Prep Kit, Illumina). The draft genome sequence of strain *S. tunisialbus* was established by sequencing on an Illumina MiSeq system. To assemble the obtained and processed reads, the GS de novo Assembler software version 2.8 (Roche, Mannheim, Germany) with default setting was applied as recently described (Yücel et al. 2017). Annotation of the draft genome was performed by applying the GenDB 2.0 system (Meyer et al. 2003) and Prokka version 1.11 (Seemann 2014). To identify clusters involved in secondary metabolite biosynthesis, the annotated genome was analyzed by applying antiSMASH 5.1.2 (Blin et al. 2017). Based on the 16S analysis in Ayed et al., *S. varsoviensis* NRRL ISP-5346 was determined as closest relative with a sequenced genome. *S. varsoviensis* NRRL ISP-5346 was found to produce multiple hygrolide sub-families, e.g., hygrobafilomycins (JBIR-100 and hygrobafilomycin) and bafilomycins (bafilomycins C1 and D) (Molloy et al. 2016). Finally, comparative genome analysis for *S. tunisialbus* DSM 105760^T^ and *S. varsoviensis* NRRL ISP-5346 was performed by means of the comparative genomics tool EDGAR (Blom et al. 2009, 2016). To determine the relatedness between the *S. tunisialbus* DSM 105760^T^ and *S. varsoviensis* NRRL ISP-5346 (Accession number: JOBF0100001-118), an average nucleotide identity analysis (ANI), average amino acid identity analysis (AAI) (Konstantinidis and Tiedje 2005) and a DDH estimation were performed as described previously (Meier-Kolthoff et al. 2013).

## Results and discussion

The MiSeq sequencing run (2 × 300 bp) resulted in 2,092,356 reads yielding approx. 627 Mb sequence information for the sample representing on average a coverage of about 90-fold. The final draft genome established by the GS Assembler software (version 2.8, Roche) consists of 35 contigs (N50: 455,831 bp) and 20 scaffolds (N50: 678,417 bp). The annotation of the draft genome of *S. tunisialbus* showed a linear chromosome which has a size of 6,880,753 bp and encodes 5802 protein-coding sequences (CDS)s. The chromosome also contains 72 tRNA genes and eight rRNA operons (16S-23S-5S rRNA). The average GC content is 71.85% (Table 1).

**Table 1:** General features of *S. tunisialbus* DSM 105760^T^ and *S. varsoviensis* NRRL ISP-5346

Features	*S. tunisialbus* DSM 105760^T^	*S. varsoviensis* NRRL ISP-5346
Length (bp)	6,880,753	8,588,807
Status	Draft genome (35 contigs)	Draft genome (118 contigs)
G + C content (%)	71.85	72.40
CDS	5802	7229
rRNAs operons	8	NA
tRNAs genes	72	68

An ANI value of 84.93%, an AAI value of 87.43% and a DDH value of 25.00% were calculated for *S. tunisialbus* DSM 105760^T^ and *S. varsoviensis* NRRL ISP-5346. High ANI, AAI values (above 95%) and high DDH estimations (Formula 2 above 70%) were usually observed for bacterial isolates representing the same species and are considered to indicate bacterial species demarcation previously (Konstantinidis and Tiedje 2005). Therefore, both strains are closely related, but do not belong to the same species.

By applying EDGAR, a comparative analysis was performed for *S. tunisialbus* DSM 105760^T^ and *S. varsoviensis* NRRL ISP-5346. It appeared that 3830 genes corresponding to 66% and 55% of all genes identified in both draft genomes represent the core set of genes shared by both isolates. These core genes mainly encode for housekeeping functions, e.g., glycolysis. However, *S. tunisialbus* DSM 105760^T^ possesses 1972 singletons, whereas 3062 singletons were identified for *S. varsoviensis* NRRL ISP-5346. Most of the *S. tunisialbus* DSM 105760^T^ singletons were annotated as ‘hypothetical’ genes or transposases. However, genes encoding enzymes potentially involved in the synthesis of secondary metabolites were identified within the unique genes of *S. tunisialbus* DSM 105760^T^. In addition, a few unique genes encoding for different cytochromes were found. For *S. varsoviensis* NRRL ISP-5346, the clusters found to produce hygrobafilomycins (JBIR-100 and hygrobafilomycin) and bafilomycins (bafilomycins C1 and D) were unique.

To identify all clusters involved in secondary metabolite biosynthesis, the *S. tunisialbus* DSM 105760^T^ genome was analyzed by means of antiSMASH 5.1.2. The platform revealed the presence of 34 predicted gene clusters encode for potential secondary metabolites, such as antibiotics, antitumor compounds, fungicide, and lantibiotics. Likewise, six of these gene clusters are terpenes, such 2-methylisoborneol [100%] a volatile organic compound well known for its biological activity, two for melanin biosynthesis and one for Ectoine (Table 2). *S. varsoviensis* NRRL ISP-5346 has a similar portfolio of secondary metabolite clusters (Table 3).

**Table 2:** Secondary metabolite cluster of *S. tunisialbus* DSM 105760^T^

Region	Type	Most similar known cluster	
Region 1	NRPS-like,lassopeptide	Citrulassin B	100.00%
Region 2	Terpene	2-Methylisoborneol	100.00%
Region 3	T2PKS, T1PKS, NRPS, terpene	JBIR-76/JBIR-77	68.00%
Region 4	NRPS	C-1027	7.00%
Region 5	Terpene	Calicheamicin	2.00%
Region 6	Ectoine	Ectoine	100.00%
Region 7	Lanthipeptide	SapB	75.00%
Region 8	Linaridin	Pentostatine/vidarabine	12.00%
Region 9	Melanin	Melanin	28.00%
Region 10	Melanin	Melanin	28.00%
Region 11	Terpene	Aristeromycin	6.00%
Region 12	Lassopeptide	Lagmysin	60.00%
Region 13	T3PKS	Herboxidiene	5.00%
Region 14	T3PKS, ladderane	Vazabitide A	28.00%
Region 15	Butyrolactone	Griseoviridin/fijimycin A	8.00%
Region 16	T1PKS	Elaiophylin	8.00%
Region 17	T1PKS	Guadinomine	7.00%
Region 18	T1PKS, thiopeptide, LAP, NRPS-like, terpene, NRPS	Aureothin	100.00%
Region 19	hglE-KS	a201a	5.00%
Region 20	Terpene	2-Methylisoborneol	75.00%
Region 21	hglE-KS, T3PKS	Alkylresorcinol	100.00%
Region 22	NRPS, T1PKS, terpene	Althiomycin	100.00%
Region 23	Bacteriocin, NRPS	Kirromycin	16.00%
Region 24	T1PKS, Terpene, bacteriocin	Nystatin A1	63.00%
Region 25	NRPS	Deimino-antipain	66.00%
Region 26	NRPS, terpene, T1PKS, transAT-PKS, PKS-like	Netropsin	100.00%
Region 27	Siderophore	Vibrioferrin	18.00%
Region 28	Terpene	Asukamycin	4.00%
Region 29	Terpene	Hopene	69.00%
Region 30	Indole	AT2433-A1	14.00%
Region 31	Butyrolactone		
Region 32	NRPS	Bacillibactin	46.00%
Region 33	T1PKS, NRPS, ladderane, arylpolyene, NRPS-like	Atratumycin	55.00%
Region 34	siderophore

**Table 3:** Secondary metabolite cluster of *S. varsoviensis* NRRL ISP-5346

Region	Type	Most similar known cluster	
Region 1	Terpene, T1PKS, NRPS-like, phosphonate, nucleoside, lanthipeptide	Phosphinothricintripeptide	17.00%
Region 2	NRPS-like, T1PKS	Miharamycin A/miharamycin B	40.00%
Region 3	PKS-like	A-201A	12.00%
Region 4	Terpene		
Region 5	NRPS	Isocomplestatin	50.00%
Region 6	Lanthipeptide	AmfS	80.00%
Region 7	Lassopeptide	Anantin B1/anantin B2	40.00%
Region 8	Furan, NRPS	Ficellomycin	3.00%
Region 9	NRPS-like, NRPS	Friulimicin A/friulimicin B/friulimicin C/friulimicin D	27.00%
Region 10	Amglyccycl		
Region 11	NRPS	Streptobactin	47.00%
Region 12	Amglyccycl	Hygromycin A	51.00%
Region 13	Fused, NRPS-like, TfuA-related, NRPS, T1PKS	Thiovarsolin A/thiovarsolin B/thiovarsolin C/thiovarsolin D	83.00%
Region 14	NRPS	rimosamide	42.00%
Region 15	T2PKS,PKS-like, oligosaccharide, LAP	Dutomycin	36.00%
Region 16	Bacteriocin		
Region 17	Terpene		
Region 18	hglE-KS, T1PKS	Tetronasin	9.00%
Region 19	Lanthipeptide		
Region 20	Ectoine	Ectoine	100.00%
Region 21	NRPS, T2PKS	Kosinostatin	6.00%
Region 22	LAP, thiopeptide	Jomthonic acid A/jomthonic acid B/jomthonic acid C	5.00%
Region 23	Thiopeptide, LAP		
Region 24	Terpene	Ansamitocin P-3	4.00%
Region 25	NRPS,CDPS	Purincyclamide	100.00%
Region 26	T1PKS	Chlorizidine A	57.00%
Region 27	Lanthipeptide		
Region 28	NRPS, T3PKS	Feglymycin	42.00%
Region 29	T1PKS, NRPS, aminocoumarin	Bafilomycin B1	83.00%
Region 30	CDPS		
Region 31	Terpene	Ebelactone	5.00%
Region 32	Siderophore		
Region 33	Siderophore	Ficellomycin	3.00%
Region 34	Siderophore		
Region 35	Terpene	Hopene	61.00%
Region 36	Bacteriocin		
Region 37	NRPS		

The presence of a large number of cryptic secondary metabolite gene clusters in its genome, offers unsuspected potential for synthesizing bioactive compounds. In addition, our study shows that sequencing of new strains allows the discovery of new secondary metabolites relevant in ecology, biotechnology and medicine.

## Data Availability

The scaffolds of the whole-genome shotgun project have been deposited in the EMBL database (EBI) under the accession no. OKRJ01000001-OKRJ01000020.
